# The metabolic vascular syndrome - guide to an individualized treatment

**DOI:** 10.1007/s11154-016-9345-4

**Published:** 2016-03-09

**Authors:** Markolf Hanefeld, Frank Pistrosch, Stefan R. Bornstein, Andreas L. Birkenfeld

**Affiliations:** GWT-TU Dresden GmbH, Fiedlerstr. 34, 01307 Dresden, Germany; Medizinische Klinik 3, Universitätsklinikum Carl Gustav Carus, Fetscherstr. 74, 01307 Dresden, Germany; Section of Diabetes and Nutritional Sciences, Rayne Institute, Denmark Hill Campus, King’s College London, London, UK; Paul Langerhans Institute Dresden of the Helmholtz Center Munich at University Hospital and Faculty of Medicine, TU Dresden, Dresden, Germany; German Center for Diabetes Research (DZD e.V.), Neuherberg, Germany

**Keywords:** Metabolic syndrome, Type 2 diabetes, Treatment, OAD

## Abstract

In ancient Greek medicine the concept of a distinct syndrome (going together) was used to label ‘a group of signs and symptoms’ that occur together and ‘characterize a particular abnormality and condition’. The (dys)metabolic syndrome is a common cluster of five pre-morbid metabolic-vascular risk factors or diseases associated with increased cardiovascular morbidity, fatty liver disease and risk of cancer. The risk for major complications such as cardiovascular diseases, NASH and some cancers develops along a continuum of risk factors into clinical diseases. Therefore we still include hyperglycemia, visceral obesity, dyslipidemia and hypertension as diagnostic traits in the definition according to the term ‘deadly quartet’. From the beginning elevated blood pressure and hyperglycemia were core traits of the metabolic syndrome associated with endothelial dysfunction and increased risk of cardiovascular disease. Thus metabolic and vascular abnormalities are in extricable linked. Therefore it seems reasonable to extend the term to metabolic-vascular syndrome (MVS) to signal the clinical relevance and related risk of multimorbidity. This has important implications for integrated diagnostics and therapeutic approach. According to the definition of a syndrome the rapid global rise in the prevalence of all traits and comorbidities of the MVS is mainly caused by rapid changes in life-style and sociocultural transition resp. with over- and malnutrition, low physical activity and social stress as a common soil.

## Introduction - the beginning

**Fig. 1:**
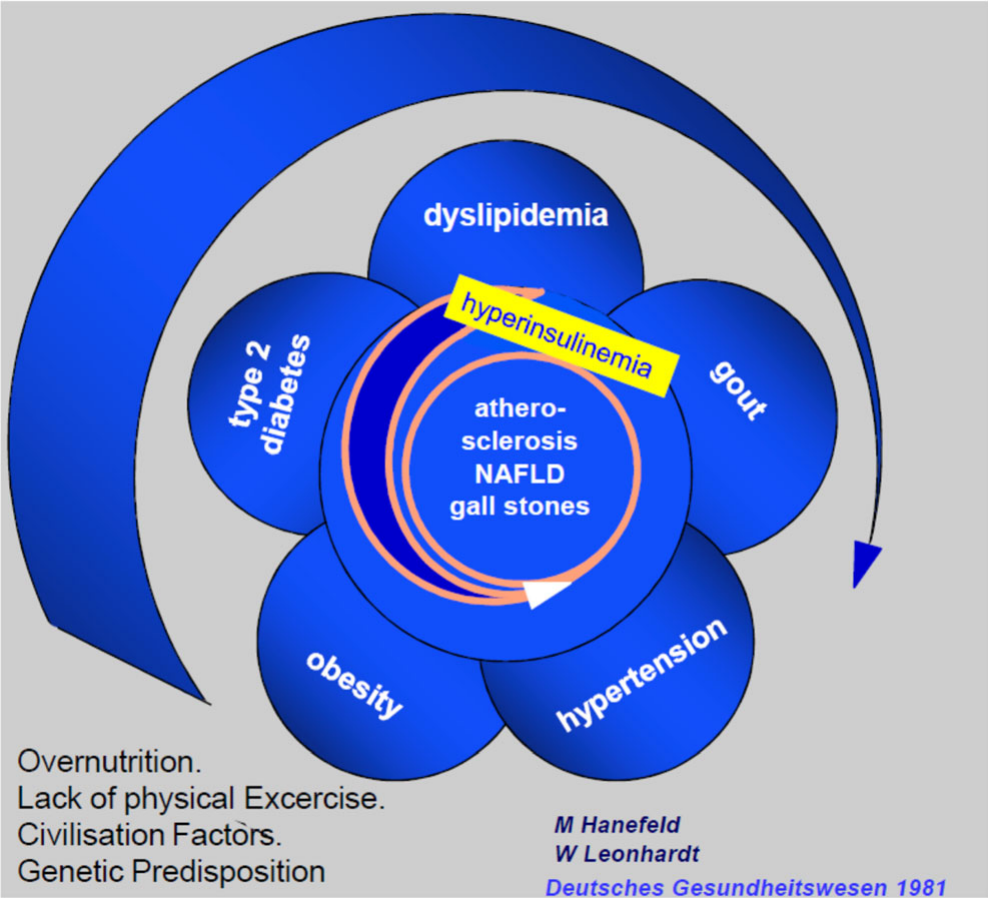
Historic vicious cycle of the metabolic syndrome [9]

The coincidence of diabetes, hypertension and gout as a syndrome has first been described in the early twenties of the last century [1, 2]. After world war II. J. Vague [3] was the first to investigate the links between android (visceral) obesity, dyslipidemia, glucose intolerance, hyperuricemia and cardiovascular disease - a condition that was later called plurimetabolic syndrome by Avogaro and Crepaldi [4]. The “second Industrial Revolution” in the mid-19th century with changes in socioeconomic structure, food supply sources and dramatic decrease in physical activity resulted in a pandemic of obesity [5]. Therefore obesity was recognized as a driving force to development of the metabolic syndrome with insulin resistance and impaired lipolysis of adipose tissue as central pathophysiology [6]. A close link of diseases of the metabolic syndrome to non-alcoholic fatty liver (NAFLD) was first recognized in the sixties [7, 8] when the Menghini technique was widely used for liver biopsies. Based on these comprehensive investigations we further worked out the concept of the metabolic syndrome as an integrated approach for diagnostic and therapy of this cluster of diseases: “*The metabolic syndrome represents the common prevalence of obesity*, *hyper- and dys-lipoproteinaemia*, *maturity onset diabetes* (*type 2*), *gout and hypertension associated with increased incidence of atherosclerotic vascular disease*, *fatty liver and gallstones that develops on the basis of genetic susceptibility combined with over-nutrition and physical inactivity. If this working hypothesis can be confirmed it provides the basis for integrated diagnostics and prevention of this cluster of diseases which is of central importance for health care.*” [9]. In a vicious cycle this develops to type 2 diabetes and atherosclerotic vascular disease (Fig. 1).

Insulin resistance as a major underlying pathophysiology for a common type of diabetes has first been described by Himsworth in 1936 [10]. In 1979 de Fronzo et al. introduced the glucose clamp technique to measure insulin-resistance *in vivo* [11]. With this new technique it could be shown that the 5 traits of the MS and atherosclerotic vascular disease are associated with hyperinsulinemia and insulin resistance [12]. In his Banting lecture G. Reaven defined therefore the insulin resistance syndrome – syndrome X – as the association of insulin resistance/hyperinsulinemia, glucose intolerance, dyslipidemia and hypertension [13].

With the pandemic of diseases of the metabolic syndrome and a plethora of studies on the syndrome X or metabolic syndrome after de Fronzo’s, Ferrannini’s and Reaven’s publications a working group of the WHO published a definition and for the first time with cut-off limits for the traits of the metabolic syndrome under the guidance of K.G. Alberti [14]. This definition and diagnosis worked out by diabetologists was primarily based on insulin resistance syndrome as the central pathophysiology (Table 1). During the following years thousands of papers have been published on links to and risk of cardiovascular disease related to the metabolic syndrome [17–19]. Based on this plethora of epidemiological investigations the AHA and ADA developed a further definition with modified cut-off limits and with the aim to have a simple guide for clinicians to diagnose people at high risk for cardiovascular disease and type 2 diabetes (Table 1) [15]. However different phenotypes of MS have not the same significance as cardiovascular risk factors [17]. Thus, traits of the metabolic syndrome cannot be used to replace established risk engines such as Framingham, PROCAM or UK-PDS risk score [20, 21]. Furthermore other definitions, changes in cut-off limits of traits and regional and ethnic differences in diagnosis of obesity made it difficult to evaluate risk for cardiovascular disease and type 2 diabetes associated with single traits, combinations or overall metabolic syndrome. Therefore, in a critical appraisal some leading diabetologists suggested: that insulin resistance is not the only unifying causal factor, the CV-risk associated with the overall metabolic syndrome is not greater than the sum of single components and not at least cut-off limits of traits are arbitrary [22]. Thus, they concluded that ‘the medical value of diagnosing the syndrome is unclear’. Since the authors of this harsh critical appraisal did not consider the very simple concept of a syndrome to have a practical guide for an integrated approach of diagnosis and treatment ‘of signs and symptoms that occur together with a particular abnormality and conditions’ the syndrome survived and still is widely used in daily practice. In 2009 a unified worldwide definition has been accepted and published by the IDF [15]. As a matter of fact in 2014 6090 and in 2015 5524 papers have been published related to the metabolic vascular syndrome and its co-morbidities.

**Table 1:** Definitions of the metabolic syndrome [14–16]

	AHA/NCEP III	IDF	Consensus statement
Central obesity/waist	>102 cm (m) >88 cm (w)	≥94 cm (m, European) ≥90 cm (m, Asian) ≥80 cm (w)	Population and county specific increased waist circumference
Blood pressure (mmHg)	≥130/85 or treated for hypertension	≥130/85 or treated for hypertension	≥130/85 or treated for hypertension
Triglycerides (mmol/l) (mg/dl)	≥1.7 (150)	≥1.7 (150) or treatment	≥1.7 (150) or treatment
HDL-cholesterol (mmol/l)/(mg/dl)	<1.04 (40) (m), <1.29 (50) (w)	<1.04 (40) (m), <1.29 (50) (w) or treatment	<1.03 (40) (m), <1.29 (50) (w) or treatment
Fasting plasma glucose (mmol/l)/(mg/dl)	≥6.1 (110)	≥5.6 (100) or diagnosed with diabetes mellitus	≥5.6 (100) or drug treatment for elevated glucose

*m* men, *w* women

## Pathophysiology: common soil and links to diabetes and cardiovascular disease

The metabolic syndrome rose to increased clinical consideration and scrutiny together with the worldwide epidemic of obesity and diabetes mellitus. However, the pathophysiological mechanisms leading to cluster of metabolic diseases and eventually cardiovascular damage are not completely understood [23]. Although insulin resistance is a core abnormality of individuals with metabolic syndrome [24], there is no sufficient evidence for a causal link between the two [25]. The most promising hypothesis for a causal link between the development of the different traits of the metabolic syndrome and atherosclerosis is chronic low grade inflammation, particularly in dysfunctional adipose tissue [26]. The onset of abdominal obesity is central to the alteration of normal adipose tissue function with decreased glucose uptake, increased storage of fat as well as increased release of non esterified fatty acids (FFA) into the circulation. In obesity adipose tissue is infiltrated by macrophages which influence its cytokine production. There is an increased release of interleukin 6, tumour necrosis factor α (TNFα), monocyte chemo-attractant protein 1 (MCP1) or C-reactive protein (CRP) whereas release of anti-inflammatory cytokines i.e. adiponectine or interleukin 10 is decreased. Whether the inflammatory response of the visceral adipose tissue is primarily induced by intracellular fat accumulation or by infiltration of activated macrophages is still a matter of debate [27]. However, recent studies in animals and cell cultures demonstrated an intensive cross talk between immune cells, macrophages and adipocytes in the generation of an inflammatory response [28, 29]. Thus, the impact of changes in visceral adipose tissue can be summarized as a state of systemic lipotoxicity and low grade inflammation. Inflammatory cytokines are involved in the induction of endothelial dysfunction and insulin resistance [30]. Furthermore the insulin resistant state of obesity is characterised by increased plasma levels of free fatty acids that have cardiotoxic effects and impair the production of endothelial vasodilators [31, 32].

In addition to these systemic effects of visceral obesity there is a local impairment of cardiac and vascular function by dysfunctional perivascular adipose tissue (PVAT) [33]. Under normal conditions PVAT produces different cytokines and hormones which contribute to vascular relaxation. In the obese state PVAT mass, like visceral adipose mass is increased and its vasodilating effects are diminished. Therefore PVAT in obesity may contribute to endothelial dysfunction and hence atherosclerosis and plays a key role in the development of vascular insulin resistance [34, 35].

A common hypothesis describes metabolic susceptibility as central factor for the development of the metabolic syndrome. This metabolic susceptibility is determined by polygenic variability of individuals [36] but also gene-environment interactions [30, 37]. Once a sedentary lifestyle with decreased physical activity and high caloric diet leads to the acquisition of body fat and to development of overweight and obesity, a susceptible individual is at high risk to develop the metabolic syndrome and cardiovascular consequences. Genome wide association studies have identified a lot of potential genetic variants that may contribute to development of metabolic syndrome. However, the complexity of its different single traits with their own genetic determinants is a major challenge for genetic studies [38]. Despite this complex pathophysiology as soil for the metabolic syndrome and associated diseases, we also have to keep in mind the strong impact of lifestyle and environment which lead to epigenetic regulation such as the methylation of DNA nucleotides and the modification of histone proteins surrounding the DNA double helix. These mechanisms as key regulators of gene expression can explain inter-individual variation of phenotypes [39]. Recent studies demonstrated a close relationship between intrauterine growth retardation and metabolic disease in adulthood. Low birth weight has also been associated with hypertension and susceptibility to cardiovascular disease [40]. In addition to heritable regulation of the epigenome, there is also evidence of lifestyle-related modification of genes in adulthood [41].

**Fig. 2:**
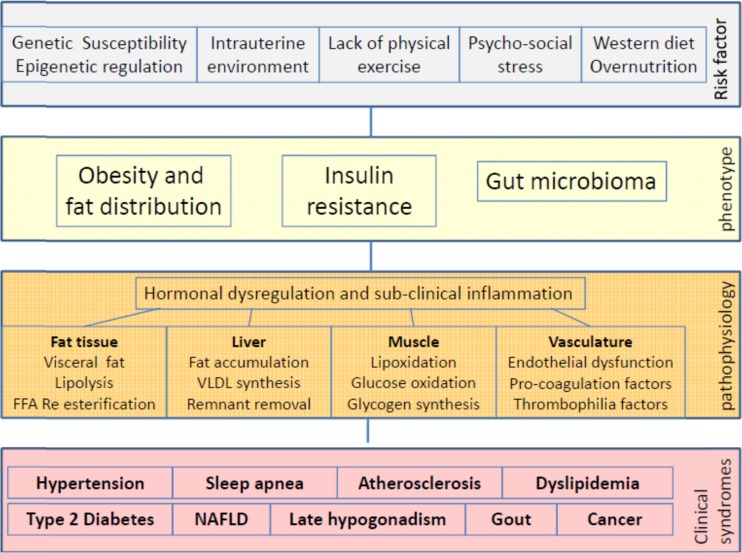
Causes and diseases of the metabolic syndrome today

As a conclusion, there are several genetic and environmental factors which contribute to the development of both metabolic disorders which are summarized as metabolic syndrome and cardiovascular disease. It is conceivable that metabolic and cardiovascular disorders develop in parallel and can influence each other. Therefore the term metabolic-vascular syndrome might be the most comprehensive description of this cluster of disease (Fig. 2).

## Individualized treatment of single components of the metabolic syndrome as prerequisite for the improvement of cardiovascular outcome

### Lifestyle intervention

From a clinical point of view type 2 diabetes and cardiovascular disease such as coronary heart disease, cerebrovascular disease and peripheral arterial disease can be considered as end-stage diseases developing on the complex prodiabetic and proatherogenic soil of the metabolic vascular syndrome. This concept is the essential basis for lifestyle intervention and improving socio-economic conditions, avoiding stress exposure with its hormonal derangements, and regulation of food production and trade [42–44].

So far best evidence for modifiable risk factors for prevention of the metabolic syndrome is available for changes in nutrition to reduce overweight and insulin resistance and increased physical activity. There exists now a bulk of evidence that with effective lifestyle intervention incidence of type 2 diabetes can be reduced by about 50 % [45, 46]. Life style intervention with similar integrated approach some of them also including psychosomatic treatment tools - have also successfully been performed for the prevention of cardiovascular diseases [47, 48]. The basic principles of life style intervention are - according to the common soil hypothesis - identical for all traits of the metabolic vascular syndrome.

### Antihypertensive drugs

International guidelines recommend systolic blood pressure control to a level < 140 mmHg and diastolic blood pressure control to <90 mmHg depending on age, individual risk and co-morbidities with focus on kidney disease [49]. These blood pressure goals might be challenged by a recent trial in patients with increased cardiovascular risk but without diabetes which demonstrated that a systolic blood pressure target of 120 mmHg was associated with fewer cardiovascular end points than a the widely recommended target of 140 mmHg [50]. Most of these patients fulfilled the definition of metabolic syndrome.

To reach the treatment goals 4 classes of antihypertensive drugs are recommended as first line treatment by the European Society of Hypertension/European Society of Cardiology (ESH/ESC) guidelines: ACE-inhibitor/ARB, calcium channel blockers, betablockers and diuretics. While blood pressure lowering effect and cardiovascular benefit is similar for these for classes of antihypertensive agents there are some differences in metabolic effects which should be considered in patients with the metabolic syndrome. Betablocker can increase body weight and – in combination with diuretics - the incidence of type 2 diabetes [51, 52]. However, newer betablocker e.g. nebivolol and carvedilol did not affect insulin sensitivity and should therefore be preferred in patients with the metabolic syndrome [53, 54]. The Avoiding cardiovascular events in combination therapy in patients living with systolic hypertension (ACCOMPLISH) trial demonstrated a higher rate of cardiovascular events in patients receiving a combination therapy of a thiazide diuretic and an ACE inhibitor compared to patients with an ACE inhibitor/calcium channel blocker [55] but no other randomized trials demonstrated this superiority of calcium channel blocker over a diuretic treatment [49]. The use of thiazide diuretics can induce hypokalemia which may worsen glucose tolerance and provoke cardiac arrhythmias [56]. Due to their unfavourable metabolic effects betablockers and diuretics should only be considered as additional blood pressure lowering drugs in metabolic syndrome. If thiazide diuretics are used the addition of an potassium sparing diuretic agents could reduce the risk of hypokalemia [57].

ACE-inhibitor or ARB and calcium channel blockers should be preferred for the treatment of hypertension in patients with the metabolic syndrome because they did not influence insulin sensitivity or body weight. ACE-inhibitors/ARB are most effective in reducing proteinuria and preventing the progression of diabetic nephropathy whereas calcium channel blockers are the best choice for the prevention of stroke [58, 59]. There is no evidence of an additional benefit of the newer ARB compared to ACE-inhibitors in patients with the metabolic syndrome.

In the ONTARGET study ARB telmisartan was associated with a significantly higher incidence of diabetes while no effect on primary objectives – major cardiovascular events was achieved [60]. In the HOPE study, however, with ACE inhibitor ramipril less patients were diagnosed with diabetes at the end of the study as in the placebo group [61]. This could not be confirmed in the DREAM trial in people with impaired glucose tolerance where ramipril had no effect on the incidence of diabetes as a primary objective [62]. The same applies for ARB valsartan in the NAVIGATOR trial - a prospective primary prevention study with cardiovascular complications as primary objective and diabetes as secondary objective [63].

To achieve blood pressure goals most patients need a combination therapy of 2 or more antihypertensive drugs. As recently recommended by the ESH/ESC Guidelines the initiation of a combination therapy instead of a monotherapy should be considered in patients with a blood pressure > 160 mmHg systolic and/or >100 mmHg diastolic because of the prompter response in a larger number of patients the greater probability of achieving target blood pressure and a higher adherence of patients to the therapy [49]. In conclusion national and international guidelines recommend in patients with the metabolic syndrome an individualized approach considering age, co-morbidities and presence or absence of end stage diseases to guide decision making.

### Antidiabetic drugs

Evidence with antidiabetic drugs for the prevention of type 2 diabetes in people with abnormal glucose tolerance is available only for metformin [64], acarbose [65] and thiazolidinediones [66, 67] and the combination of metformin plus rosiglitazone [68].

Metformin has consistent evidence to prevent progression of IGT/IFG to type 2 Diabetes. In the DPP Study the reduction in incidence of diabetes was 31 % vs. life style intervention alone [64]. The reduction of newly diagnosed diabetes in the STOP NIDDM with α glucosidase inhibitor acarbose was in the same range if diagnostic criteria were used as in the DPP [65]. Despite glitazones were very effective to reduce incidence of newly diagnosed diabetes and had pleiotropic effects on blood pressure, biomarkers of inflammation and endothelial dysfunction [69–72] they cannot be recommended because of serious adverse events such as edema, congestive heart failure and bone fractures for primary prevention of diseases of the metabolic syndrome [73]. Orlistat, a weight reducing intestinal lipase inhibitor reduced incidence of diabetes in obese subjects with abnormal glucose tolerance by ~31 % [74]. Metformin in addition had beneficial effects on weight and minor effects on blood lipids, but did not affect blood pressure in the DPP [75] and BIGPRO trial [76]. However, none of the primary prevention trials with metformin has shown an effect on major cardiovascular events also in the long term follow-up after termination of the studies with a duration of ~3 years. Acarbose so far is the only antidiabetic drug with a significant pleiotropic effect on elevated blood pressure [77]. It significantly reduces body weight, postprandial hyperinsulinemia, biomarkers of inflammation and hypertriglyceridemia [78, 79]. Predefined cardiovascular events were secondary objectives in the STOP-NIDDM trial. In this trial a significant reduction in the incidence of myocardial infarction and of cardiovascular events was registered [77]. Furthermore 36 % less newly diagnosed cases of hypertension were observed. Of notice stable IGT or remission to NGT was associated with a lower incidence of hypertension compared to progression to type 2 diabetes [80]. Intervention with basal insulin glargine in prediabetic subjects was evaluated in the ORIGIN trial. Reduction of newly diagnosed diabetes 3 months after stopping insulin treatment was 20 %. There was, however, no effect on major cardiovascular events achieved [81].

### Lipid lowering drugs

Dyslipidemia with hypertriglyceridemia and low HDL is in the majority of cases associated with an increase in small dense LDL a lipoprotein fraction with high atherogenic potential which is intricately connected with insulin resistance and low grade inflammation [82]. This lipid triad together with high cardiovascular risk provides a rational pathophysiological basis for the use of statins as first line drug [83–86]. As shown in a meta-analysis of data from 170,000 participants with intensive statin treatment reduction of cardiovascular events was mainly due to LDL-cholesterol lowering efficacy [87]. Beneficial effects on the lipid triad in patients with the metabolic syndrome have been documented for atorvastatin and rosuvastatin [88, 89]. However, in long term studies some of the more potent statins increased the risk of newly diagnosed diabetes [90]. This is far outweighed by the cardiovascular benefit. In a meta-analysis intensive dose statin therapy had a number needed to harm for one case of new onset diabetes of 498 versus a number needed to prevent one case of major cardiovascular events of 155 per year [90]. In addition statin treatment has a small but significant beneficial effect on blood pressure [91].

Fibrates have been shown to reduce cardiovascular events in patients with the metabolic syndrome and type 2 diabetes when added to a statin therapy [92, 93]. However, fibrates in combination with statins can increase the rate of myopathy and risk of rhabdomyolysis [94]. The concept to increase HDL-cholesterol to protect the vessel wall was not supported by recently stopped trials with nicotinic acid [95] and CETP inhibitors [96, 97] showing increased rates of serious adverse events. Therefore ESC Guidelines no longer support drug interventions to increase HDL-cholesterol [98]. Newer drugs such as ezetimibe - an inhibitor of the intestinal cholesterol absorption that may also improve traits of the metabolic syndrome [99] - or the humanized antibody against proproteinconvertase subtilisin/kexin type 9 (PCSK9) can significantly reduce LDL-cholesterol in combination with statins. However, an improvement of cardiovascular end points with ezetimibe was restricted to patients with an acute coronary syndrome [100] and there is still a lack of results regarding cardiovascular end points from studies with PCSK9 inhibitors.

### Anticoagulant therapy

Patients with a metabolic syndrome have a complex pathophysiology of cellular and humoral coagulation with activated platelet aggregation, impaired fibrinolysis and elevated factors of the coagulation cascade as major components. This is particularly critical in patients with type 2 diabetes [101–103]. Subjects with diabetes have a higher rate of major cardiovascular events but lower efficacy of intervention after acute coronary syndrome [104]. This can be at least partially explained by harmful alterations in the coagulation associated with the metabolic syndrome. According to this critical weight of atherothrombogenic risk factors randomised trials and meta-analysis revealed a greater benefit of anticoagulatory prevention for patients with diabetes and metabolic syndrome.

Acetyl salicylic acid (aspirin) is widely used for primary and secondary prevention in type 2 diabetes. Recently published meta-analysis, however, reveal no significant impact on mortality while bleeding episodes are significantly increased [105, 106]. No data on primary prevention are available for the new platelet aggregation inhibitors such as clopidogrel, prasugrel and ticagrelor. The benefit of low dose aspirin (75–100 mg/d) for secondary prevention is well documented for patients with type 2 diabetes [105]. In the CAPRIE study clopidogrel 74 mg was significantly more effective in patients with type 2 diabetes compared to ASS [107]. Incidence of MACE with clopidogrel was 5.32 %, with aspirin 5.83 % (RR 8.7 %, *p* = 0.043). Benefit of clopidogrel was even higher in patients with peripheral arterial disease. Therefore, the ADA recommends clopidogrel in very high risk groups with type 2 diabetes. This could be applied in general for type 2 diabetes and metabolic syndrome. New platelet aggregation inhibitors prasugrel and ticogrelor have shown a significantly higher benefit in acute coronary syndrome versus clopidogrel [108]. However, large outcome trials in patients with stable atherosclerotic disease are not yet published. Diabetes is an independent risk factor for atrial fibrillation and thrombolic complications. Therefore risk scores for stroke and systemic embolism result in an indication for anticoagulant therapy with vitamin K antagonists (cumarins) or direct-acting oral anticoagulants (apixaban, dabigatran, rivaroxaban). No data from controlled prospective trials are available in diabetes comparing old and new anticoagulants.

### Bariatric surgery

Abdominal obesity is the most common single trait of the metabolic syndrome [17] and central in the pathogenesis of cardiovascular events and type 2 diabetes. However, a durable sufficient weight loss is uncommon with medical or lifestyle approaches and adequate glycemic control often remains elusive [109]. Therefore, bariatric surgery also regarded as “metabolic” surgery due to its favourable effects on the metabolic syndrome is now increasingly used to achieve a sustained weight loss and a regression of type 2 diabetes [110, 111]. Further effects after bariatric surgery were lowering of systolic and diastolic blood pressure and improvement of the lipid profile. The detailed description of different operation techniques is beyond the scope of this review however, there are also data about resolution of the metabolic syndrome after bariatric surgery: Batsis and co-worker described a decrease of the prevalence of the metabolic syndrome from 87 to 29 % before vs. 3.4 years after gastric bypass whereas the prevalence in the control group treated with lifestyle intervention decreased from 85 to 75 % within the same time period [112]. The remission rate of the metabolic syndrome was significantly higher after malabsorptive surgery i.e. gastric bypass or biliopancreatic diversion compared to techniques which mainly restrict energy intake (gastric banding or sleeve gastrectomy) [113]. The profound resolution of metabolic deteriorations and the resulting reduction of mortality at least in patients with type 2 diabetes [114] lead to the extension of the indication of bariatric surgery over time: most recently even adolescents (mean age 17 ± 1.6 years) with obesity stage 3 (mean BMI 54 kg/m^2^) underwent gastric bypass or sleeve gastrectomy with favourable effects regarding weight, dyslipidemia, blood glucose and blood pressure control [115]. Nevertheless these invasive techniques are not free of adverse events which include the need for supplementation of micronutrients or the risk of additional operative procedures. Before surgery, physiological maturation (puberty) and adequate psychological maturity should be documented [116].

## Emerging concepts in the pathophysiology of the metabolic vascular syndrome

### Substrate flux

Novel concepts link adipose tissue macrophage infiltration with substrate flux to the liver, resulting in hyperglycemia, hyperinsulinemia and dyslipemia. The fast suppression of hepatic glucose production after meals within minutes seems not to be mediated by a direct transcriptional effect of insulin in the liver [117]. Recent studies suggested that insulin action in white adipose tissue rather is the rate limiting step, lowering fatty acid and glycerol mobilization resulting in reduced hepatic acetyl-CoA content, which is an allosteric activator of pyruvate carboxylase and thus, hepatic glucose production [118]. In the setting of obesity and the metabolic vascular syndrome, macrophage infiltration into white adipose tissue (WAT) leads to increased lipolysis via cytokines, such as interleukin-6, routing fatty acids and glycerol to the liver. Here, these substrates promote fasting and postprandial hyperglycemia by augmenting hepatic acetyl- CoA content, activating pyruvate carboxylase and eventually resulting in increased glycerol conversion to glucose. [118, 119]. Moreover, increased adipose tissue lipolysis stimulates hepatic triglyceride synthesis and hyperlipidemia due to increased fatty acid esterification. These mechanisms foster an environment of excessive postprandial hyperglycemia and dyslipidemia, the cornerstones of the metabolic vascular syndrome. Therapies that decrease ectopic lipid storage [120] and diminish macrophage induced WAT lipolysis [118] will be able to reverse some of the root causes of type 2 diabetes and the metabolic syndrome.

### Gut mircobiota

Obesity and type 2 diabetes are characterized by reduced fecal microbial diversity which is linked to increased inflammation and decreased insulin sensitivity [121, 122]. Moreover, recent studies demonstrated that the consumption of high fat diets, artificial sweeteners and other dietary interventions alter the gut microbiota, resulting in intestinal inflammation and development of the metabolic syndrome [123, 124]. These findings suggest that our modern lifestyle alters the gut microbiota, and via this mechanism contributes to the complex pathophysiology of the metabolic vascular syndrome. In favor of this notion, societies with traditional lifestyles are characterized by high microbial diversity [125] and lower rates of metabolic disease. The therapeutic potential of fecal transplantation from lean donors to patients with the metabolic vascular syndrome has been examined in a pilot study. Allogenic, but not autologous, infusion of gut microbiota from lean donors into patients improved insulin sensitivity of recipients as measured with the hyperinsulinemic euglycemic clamp technique [126]. These data demonstrate that the gut microbiota is involved in the pathophysiology of the metabolic vascular syndrome. In addition, interventions helping to keep the gut microbiota healthy seem to have an effect on insulin sensitivity, and likely other components of the metabolic vascular syndrome. However, this notion still needs to be formally tested.

### Brown adipose tissue

Several research groups independently demonstrated that adults have metabolically active brown adipose tissue [127]. Its capacity to oxidize fatty acids and glucose without ATP production contributes to energy expenditure and glucose homoeostasis. In addition to classical brown adipose tissue, specific depots of inducible brown adipocytes have been identified within the white adipose depot, which are termed beige or bright adipocytes [128]. Beige cells can be induced by cold and a broad spectrum of hormones. A role of beige adipose tissue in human obesity has recently been demonstrated in elegant studies from Claussnitzer etl al. [129]. The authors show that a variant (rs1421085) in the FTO gene region, which houses one of the strongest genetic associations with obesity in genome wide association studies, dynamically modulates mitochondrial activity of human white adipose tissue, leading to reduced activation of beige adipocytes, a cellular phenotype consistent with obesity. These data raise the possibility of thermogenesis in adipose tissue as a therapeutic target for the treatment of metabolic diseases. However, an unsolved problem with the induction of beige and brown adipose tissue is the generation of heat, which can have harmful effects [130].

### Future emerging treatment strategies

#### Antidiabetic drugs

Large cardiovascular outcome trials testing newer antidiabetic drugs in patients with the metabolic vascular syndrome were conducted to provide evidence that modern therapies are as secure as classical antidiabetic therapies, i.e. metformin, sulphonylureas, insulin, thiazolidinediones and alpha-glucosidase inhibitors. Although studies with DPP-IV inhibitors [131–133] and GLP-1 receptor agonists [134] were able to provide this evidence, they failed to show superiority in terms of survival as well as macro- and microvascular complications compared to standard therapy. Recent studies with the SGLT2 inhibitor empagliflozin changed this paradigm. In the EMPA-REG outcome trial, empagliflozin 10 or 25 mg were given on top of standard therapy (including state of the art antihypertensive and lipid lowering therapy) and compared to placebo in diabetic patients with a mean waist circumference of 105 cm (BMI 30,1 Kg/m^2^) [135]. In this setting, the inhibition of SGLT2 after a median follow up of 3.1 years, resulted in superiority of empagliflozin in regards to the primary composite cardiovascular endpoint (HR = 0.86; 95 % CI 0.74-0.99; *P* = 0.04), hospitalization for heart failure (−35 %), cardiovascular mortality (−38 %) and all-cause mortality (−32 %, each *p* < 0.001). The reduction in mortality occurred rapidly (<4–6 months) and was similarly distributed in all subgroups. This reduction in mortality does not seem to be fully explained by the concomitant slight reductions in HbA1c, body weight, waist circumference and blood pressure in the empagliflozin groups versus the placebo group. It is tempting to speculate that the reduction of glucotoxicity by the pure excretion of glucose, in contrast to cellular uptake of glucose into cells, might contribute to the effect. The safety profile of empagliflozin was reasonably good. However, the FDA is currently carefully examining cases of ketoacidosis reported with SGLT2 inhibition. Future data will show if this is a concern which can be better understood and prevented when specific precaution is taken, comparable to lactic acidosis seen in rare cases with metformin. Moreover, future studies will need to show if SGLT2 inhibition is able to also prevent or delay the onset of the metabolic vascular syndrome and if it is also effective in primary prevention from micro- and macrovascular disease in type 2 diabetes. So far, the data provided by the EMPA-REG study are promising and show for the first time that a modern antidiabetic agent with effects on at least three components of the metabolic syndrome - hyperglycemia, elevated blood pressure and obesity - was able to provide superiority over standard therapy in terms of survival in a cardiovascular outcome trial [135].

#### Anti-obesity drugs

Glucagon-like peptide-1 (GLP-1) analogs are in clinical use for the treatment of type 2 diabetes. Since some of them also lead to substantial weight loss, specific GLP-1 receptor agonists (GLP-1 RA), such as liraglutide 3 mg, are now also approved in some countries for the treatment of obesity in non-diabetic patients[136]. Interestingly, in a very recent study, liraglutide 1.8 mg was also able to improve biopsy proven non alcoholic steatohepatitis [137]. So far, it is not entirely clear whether this effect is independent of body weight reduction. However, medications treating at the same time type 2 diabetes, obesity and non-alcoholic fatty liver disease, cornerstones of the metabolic vascular syndrome, appear to be attractive.

Newer attempts also aim to combine GLP-1 RAs with additional hormones. GLP-1/glucagon receptor co-agonists are currently in phase 1/2 studies for the treatment of type 2 diabetes. However, preclinical data and first clinical studies indicate a strong effect on body weight as well. Glucagon has positive effects on energy balance, body fat, and nutrient intake in rodents and humans. Oxyntomodulin, a combined GLP-1/glucagon- receptor co-agonist, reduces body weight compared with placebo and with GLP-1 agonism in obese and overweight individuals [138, 139]. Additionally, it was proven to reduce food intake after an ad libitum test meal and increase energy expenditure in humans [138]. Increased energy expenditure cannot be explained by GLP-1 RA. Additionally, co-agonists of the glucagon and GLP-1 receptor reduce cholesterol, improve insulin sensitivity and improve blood glucose levels. The effect seems to be independent of the appetite suppressing and body weight lowering effect in diet-induced obese mice [140].

Another incretin based combination is the GLP-1/GIP co-agonist. GIP shares a 37 % amino acid sequence identity with GLP-1. Because of the similarity of interaction sites, constructing single peptides with activity of both incretin hormone receptors is possible, which could result in a more pronounced antidiabetic effect. Preclinical data for GLP-1/GIP receptor agonists showed stronger reductions of blood glucose and body weight in diet-induced obese mice compared to once-daily treatment with liraglutide. However, GIP might also have negative effects on hepatic lipid content in preclinical studies [141]. More studies are needed to get a broader view on all facets of metabolic effects.

#### Lipid lowering therapy

Ideally, newer classes of lipid lowering drugs should not only treat hyperlipidemia, but also positively affect glucose metabolism in patients with the metabolic vascular syndrome, specifically because statin therapy carries a small but increased risk for type 2 diabetes. Inhibition of ATP–citrate lyase (ACL) with concomitant activation of adenosine monophosphate–activated protein kinase (AMPK) might be able to fill this medical need [142]. In type 2 diabetic patients, the ACL inhibitor bempedoic acid (ETC-1002), which in high doses can also activate AMPK, reduced LDL-C by 43 % without significant changes in triglyceride or high-density lipoprotein cholesterol. Moreover, bempedoic acid (ETC-1002) reduced high sensitivity C reactive protein (hsCRP) values by 41 %, possibly due to an immune modulating action [143]. Bempedoic acid (ETC-1002) did not affect fasting or postprandial glucose levels as measured with continuous glucose monitoring. However, in an obese subgroup of type 2 diabetic patients, it reduced daily peak and postprandial glucose levels [142]. These preliminary findings are interesting. While they do not allow to conclude that bempedoic acid (ETC-1002) has a clinically useful antihyperglycemic effect on glucose metabolism in diabetic patients at this time, it is reassuring that newer lipid lowering drugs do not aggravate glucose intolerance while clearly reducing LDL-C levels. Moreover, preclinical studies suggest that inhibition of ACL also improves non alcoholic fatty liver disease (NAFLD) [143]. It will be very interesting to see whether or not such an effect can be observed with bempedoic acid (ETC-1002) in patients with NAFLD/NASH.

#### Heart failure therapy

Individuals with diabetes and the metabolic vascular syndrome are not only at high risk of developing heart failure but are also at increased risk of dying from it. Type 2 diabetes mellitus is associated with a more than 2-fold greater risk of developing heart failure, and a 60 %–80 % greater probability of death in those with established heart failure [144]. Moreover, there has been some concern that hypoglycemic agents might contribute to a poor effect on heart failure. Therefore, strategies able to treat both conditions simultaneously are a clinical need. The cardiac hormones natriuretic peptides (NP) have been shown to have positive effects on blood pressure and cardiac function, but also to have beneficial metabolic effects, including the activation of lipolysis and energy expenditure in clinical studies and insulin sensitization and body fat reduction in preclinical studies [145–148]. Novel treatment strategies focus on inhibiting neprilysin, the neutral endopeptidase responsible for cleaving NP as well as other vasoactive hormones. This approach has been combined with the blockade of angiotensin receptors (ARB) to form LCZ696 (sacubitril/valsartan), which has recently been approved for the treatment of heart failure in some countries. LCZ696 (sacubitril/valsartan) was superior to enalapril in reducing the risk of death and of hospitalization for heart failure in the PARADIGM-HF trial [149]. Moreover, LCZ696 (sacubitril/valsartan) was also beneficial compared with enalapril in patients with type 2 diabetes and heart failure with reduced ejection fraction, irrespective of glycemic status [150]. Studies on the metabolic effects of this compound in patients with the metabolic syndrome but without heart failure are currently ongoing. Time will tell if combined ARB and neprilysin inhibition (ARNI) will be able to treat multiple components of the metabolic vascular syndrome [151].

## Conclusion

After 90 years of the first publications on close association of diabetes, hypertension and gout as a syndrome, the metabolic syndrome has been established and is widely used as a simple guide for integrated, rational diagnostics and treatment of a common cluster of metabolic vascular diseases. Over the last 5 decades the metabolic syndrome has experienced many changes in definition with a metamorphosis from a syndrome to a cluster of premorbid risk factors of cardiovascular disease and type 2 diabetes as described in the consensus statement of the IDF. According to the philosophy behind the concept of a syndrome we still see obesity, dyslipidemia, diabetes and hypertension as core components of the metabolic vascular syndrome along the continuum of prestages of these diseases. Despite critical appraisals about the pathophysiological link between its single traits, the concept of the metabolic syndrome has been proven as valuable guide for clinical decisions regarding a more precise, individualized treatment of its components and to stimulate clinical research to develop new therapies with pleiotrophic effects which target the whole cluster of associated diseases.
